# Perspectives of adult patients with mental health disorders on the relationship with nurses: a focus group study

**DOI:** 10.1186/s12912-023-01663-5

**Published:** 2024-01-02

**Authors:** Joana Coelho, Antonio Moreno Poyato, Juan Roldán Merino, Carlos Sequeira, Francisco Sampaio

**Affiliations:** 1 Research and Development Unit, Northern Health Higher School of the Portuguese Red Cross, Oliveira de Azeméis, 3720-126 Portugal; 2 Faculty of Medicine, University of Porto, Porto, 4200-319 Portugal; 3 Department of Public Health, Mental Health and Maternal and Child Health Nursing, Nursing School, Universitat de Barcelona, Barcelona, 08007 Spain; 4 NURSEARCH - 2021 SGR 01083, Mental Health, Psychosocial and Complex Nursing Care Research Group, Universitat de Barcelona, Barcelona, 08007 Spain; 5 Campus Docent Sant Joan de Déu, University of Barcelona, Barcelona, 08830 Spain; 6 CINTESIS@RISE, Nursing School of Porto (ESEP), Porto, 4200-450 Portugal; 7 Nursing School of Porto, Porto, 4200-072 Portugal

**Keywords:** Mental health, Psychiatric nursing, Patients, Focus groups, Nurse-patient relations

## Abstract

**Background:**

The relationship between the nurse and the patient with mental health disorder is crucial to the recovery process. Thus, patients with mental health disorders should be active subjects in this relationship by having autonomy and self-determination.

**Methods:**

This study aimed to explore the perspectives of adult patients with mental health disorders on the relationship with nurses. A qualitative, descriptive, and exploratory study was conducted in March 2023, using focus group meetings in an association to support patients with severe mental health disorders in the Northern region of Portugal. The study followed the Consolidated Criteria for Reporting Qualitative Research (COREQ). A total of 8 patients participated in the study. Two focus group meetings were conducted. The inductive method was used, and content analysis of the transcripts was performed. The *QDA Miner Lite 4.0* and *Microsoft Excel* were used for content analysis.

**Results:**

Participants considered the relationship with nurses important for their recovery and expected nurses to provide support and help, being able to identify their needs, thus personalising their care. Attitudinal and communication aspects were also considered crucial for establishing a solid, trusting, and meaningful relationship.

**Conclusion:**

According to the findings nursing care is expected to focus on the patient, his/her preferences, expectations, and the uniqueness of each individual. The results of this study may be useful for the reflection and improvement of nurses in their relational and communication skills and the driving force for nursing students’ awareness of the perspective of the relationship with patients with mental health disorder and its relevance.

## Background

The relationship between the nurse and the patient has long been considered a pillar in the context of mental health and psychiatric nursing [1]. Nurses are the professional group closer to patients with mental health problems [2]. In this study, the therapeutic relationship was assumed to be supportive and non-judgmental, happening in a safe environment, requiring true interest in the other, empathy, and effective communication [3].

The relationship established between the health professional providing care and the recipient of care is the most robust predictor of good treatment outcomes, regardless of the specific technique, model, or approach of the intervention [4–6]. Concerning psychotherapeutic intervention, the literature also supports this view [7].

The core of the nursing profession focuses on providing care through establishing effective and quality relationships with the recipient of care [8]. In fact, the therapeutic relationship is one of the most important working tools for nurses [9]. In nursing, the therapeutic relationship was first conceptualised by Hildegard Peplau, through the Theory of Interpersonal Relationships. This theory emphasises the patient’s experience and the impact that the relationship established with the nurse had on this experience [10], in addition to the importance of the relationship established between the nurse and the patient [11]. Peplau defined that the relationship between the nurse and the patient is established through four phases (orientation, identification, exploitation, and resolution) [12]. Through this relationship, nurses help patients improve their health status so they can develop interpersonal and problem-solving skills [13].

Concerning patients with mental health disorder, the therapeutic relationship is vital to provide quality care and promoting patient recovery [14]. In addition, the literature shows that meaningful therapeutic relationships decrease the severity of mental illness symptoms, improve quality of life and social functioning, and reduce the length of hospital stay [15].

Therefore, since a therapeutic relationship is established between nurses and patients, it is important to understand the patient’s perspective in this process, namely the patient with mental health disorder, since there has been little research in this area [16]. Thus, in this study, the experience of patients with mental health disorder is considered crucial to stimulate the delivery of high-quality care in health services [17]. This assumption is in line with the concept of co-production, in which the central idea is the collaboration between health professionals and patients, in this case, with mental disorder, so that these patients can also contribute to the management of their health condition [18]. Co-production is an interdependent work involving health professionals and their users, aimed at creating, developing, assessing, and improving actions that lead to patients’ health improvement [19]. Moreover, co-production advocates equal and reciprocal work between health professionals and patients, including in mental health contexts [20].

The literature shows several studies focussing on nurses’ or nursing students’ perceptions of the therapeutic relationship with patients [5, 21]. However, no studies were found that specifically considered the mentally ill patients’ perspective on this relationship, its importance, and what they expect from nurses concerning their behaviour and attitudes.

Despite the scarcity of research on mentally ill patients’ perspectives on therapeutic relationships, a quantitative study carried out in 12 acute mental health units in Spain aimed to examine the association and differences between nurses’ and patients’ perspectives on establishing a therapeutic relationship during the first days of hospitalization. The results indicated that patients with a diagnosis of anxiety disorder had a more positive outlook on the quality of the therapeutic relationship. Moreover, they showed that the nurse’s age had a negative impact on the patient’s perception of the quality of the therapeutic relationship [22]. However, other quantitative studies have shown that age and mental health experience are positively associated with better patient-perceived quality of the therapeutic relationship [23]. Also, a qualitative study was conducted in Spain with 11 participants who had experienced hospitalization processes in mental health units, aimed to identify the facilitating and hindering aspects of developing the reserved therapeutic space intervention model from the patient’s perspective. The results indicated that, according to the patients, the starting point for building a therapeutic relationship is the nurse’s understanding of the violation of rights often perceived by hospitalized people. Also, the nurse’s interest and empathic attitude towards the relationship and knowledge of the patient’s lived experience were highlighted [24].

The therapeutic relationship with patients with mental health disorders has been widely investigated, as evidenced by Hartley et al. [8]. However, the research primarily focuses on the perspective of health professionals, namely nurses, like in the study conducted by Tolosa-Merlos et al. [6].

Based on Hildegard Peplau’s Theory of Interpersonal Relations, in the therapeutic relationship both participants have the same value and meaning in the relationship. In light of the above, it is crucial to further the research processes that include patients with mental health disorders, as they are the central element of the relationship and health care. Person-centered care also considers the patients’ preferences, values, and needs [25].

Thus, this study sought to allow patients with mental health disorder in Portugal to express what they find crucial to their relationship with the nurse so that the nurse can act as a facilitator in the patient’s recovery process. On the other hand, this study is particularly relevant because, according to Hildegard Peplau, nursing research should focus on patients with an illness and their perception of the nursing care they receive [13]. Therefore, the purpose of this study was to highlight the perspective of patients with mental health disorders on their relationship with nurses, placing them at the centre of the investigation. In a preliminary search of national and international literature, the studies’ sampling included only nurses and not patients with mental health disorders. In addition to what has been described, this study aimed to leverage awareness of the need to involve patients with mental health disorders in research processes and consequently improve the relational skills of nurses who work with these patients.

## Aim

This study aimed to explore the perspectives of adult patients with mental health disorders on the relationship with nurses. The following research questions were formulated: What is the perspective of patients with mental health disorder on the relationship with nurses? What do patients with mental health disorders expect from this relationship, what is its importance, and what skills/attitudes are expected from nurses?

## Methods

### Design

A qualitative, descriptive, and exploratory study was conducted through focus group meetings, following Krueger and Casey [26] recommendations. The qualitative approach allows the study of the nature of phenomena, including the perspective through which they can be perceived [27]. The focus group is a technique based on the assumption that group interaction encourages participants to explore and clarify individual perspectives and share them, allowing the collection of data through group interaction on a topic suggested by the research [26]. The study followed the Consolidated Criteria for Reporting Qualitative Research (COREQ) [28].

### Selection of participants

The recruitment of participants is a systematic process; therefore, this study followed Krueger and Casey’s [26] recommendations, meaning that potential participants were contacted two weeks before the first focus group meeting to be provided with information about the study and the reasons for doing the research, and about interests in the research topic of the principal investigator. Participants were immediately informed that there would be no financial compensation for their participation. However, as a gesture of appreciation, they were also informed that at the end of each meeting there would be a shared snack time which was entirely the responsibility of the principal investigator. Furthermore, they were made aware of the importance of their involvement in the research processes and the advantages this could bring to improving care provision. Their interest in participating in the study was communicated to the association’s Vice-President, who, in turn, informed the principal investigator.

Participants were aged 18 or over, meeting the following inclusion criteria: (a) have a diagnosed mental health disorder; and (b) have previous or current contact with nurses in a clinical context. Participants were excluded if they presented: (a) severe cognition and/or communication impairment; (b) psychomotor agitation; (c) latent hostility; (d) delusional or hallucinating activity; and (e) not knowing how to read or write. Information on the exclusion criteria was provided to the vice-president of the association, who played a pivotal role in selecting participants because of prior knowledge of their clinical condition and through behavioural observation. It is important to highlight that the association’s vice-president is a mental health nurse, therefore, with the necessary knowledge and competence to carry out this assessment. It should also be noted that patients with mental health disorders can only join the association if they are psychopathologically stable.

In this type of study (focus group), the number of participants must vary between 4 and 12, and ideally between 5 and 8 [26]. However, according to Nyumba [29] there should be an over-recruitment of about 10–25% since there is no guarantee of acceptance by the potential participants.

The participants were recruited through an association for the support of patients with severe mental health disorder located in the Northern region of Portugal to include their users in the focus group since there was a high likelihood of meeting the inclusion and exclusion criteria. These criteria were presented to the Vice-President (mental health nurse), who performed a previous assessment of the users. Thus, from the 15 potential participants, 8 accepted to participate. The participants who declined the invitation were informed that their withdrawal would not result in any harm.

The potential participants were invited in person by the principal investigator at the association, in the presence of the Vice-President. Participation was voluntary and without compensation. Participants were also provided with all information about the study and asked to sign informed consent. After this stage, the focus group meetings were scheduled according to the participants’ availability. No relationship had been established with the participants prior to the beginning of the study.

Including participants with mental health disorder in this study was grounded on a logic of co-production, since in health research, bringing benefits to citizens needs to engage all users in the discussion of the research questions [30].

The focus group meetings were held in the association’s meeting room, as it is an easily accessible, offering comfort and information confidentiality, as recommended by Krueger and Casey’s [26]. Only participants and researchers were present in focus group.

### Data collection

This study included two focus group meetings. All data were collected in European Portuguese since this is the native language of the principal investigator (female sex) and participants. The principal investigator has had prior training in conducting and analysing data from focus group studies. A questioning route was previously developed to meet the study aim and research questions [31]. The questions promoted discussion, used simple terms, and were short and objective. The first questions were essentially open-ended questions, which were closed as the meeting went on, following Krueger and Casey [26] recommendations. The questioning route was previously reviewed by five patients who met the same inclusion and exclusion criteria as the study participants to ensure that all questions were clear and understandable. The script was organized as follows: (a) presentation of the researchers, (b) presentation of the study and clarification of doubts, (c) presentation of the objective of the meeting, (d) recognition and thanks for the presence and availability of the participants, (e) explanation on the meeting process, (f) confirmation of consent for audio recording and (g) exploration through questions. Concerning the questions asked, they were organized into two large groups: (a) involvement questions, such as: Would you like to introduce yourself briefly? How have you been since the last meeting?; and (b) exploratory questions, such as: Do you consider the relationship with the nurse to be of any importance? What aspects do you consider most important in the relationship with nurses? Is communication important in the relationship with the nurse? What do you think the nurse should do to communicate well with the patient with a mental health disorder? What should the nurse do/how should the nurse behave so that he or she has a good relationship with a patient with a mental health disorder?

In March 2023, the principal investigator conducted two focus group sessions supervised by another team researcher. Meetings were held face-to-face, lasting about 90 min. According to the literature, each meeting can last up to 150 min; however, ideally, it should last about 90 min [26].

Both meetings were recorded after written authorization by participants to guarantee the reliability of data analysis. Field notes are made during the focus group interview. Recordings were transcribed after each meeting using *oTranscribe*, using codes to identify participants (e.g., P1 – Participant 1, P2 – Participant 2). After this step, the transcripts were returned to the participants so they could make comments, suggestions and/or corrections. No suggestions for improvement and/or change were presented. The transcripts should portray the reality of what was discussed so that the reading enables “visualizing” the message and use this content for data analysis [32].

Thus, the characteristics of the relationship between the patient and the nurse, the contexts in which this relationship takes place, its domains, objectives, nurses’ attitudes and how these can be assessed were discussed with the participants until no new information was produced and a clear pattern of answers was achieved.

### Data analysis

Data analysis from focus group meetings must be systematic and rigorous [26]. Although there are several approaches to qualitative data analysis, it is commonly organised into three moments: (1) coding - a process of assigning categories after several readings of the transcript, reflecting the areas of the script or new areas that may have emerged; (2) storage - a compilation of text extracts associated with the same category; and (3) interpretation - a systematic analysis of the data [32].

The inductive method was used, and content analysis of the data extracted from the study was performed; therefore, the recording transcript was a unit of analysis [33]. The *QDA Miner Lite version 4.0* and *Microsoft Excel* were used for content analysis. Firstly, the principal investigator performed several readings of the transcript document for an overview of all the information. The text was then divided into units of meaning to be condensed and coded. The similarities and differences between the codes allowed for identifying categories which, by their interpretation, originated themes [33, 34]. This process was conducted by the principal investigator and then checked by the other research team members. Three meetings were held for discussion and critical reflection, with consensus reached throughout this process. Because of the abundance of data obtained after the second group, it was decided that the two groups were sufficient to achieve the study’s objective beyond obtaining data saturation [35]. The results were presented to the participants, who gave a positive opinion towards them.

### Rigour

The rigour of the study was based on credibility, transferability, dependability, and confirmability measures [36]. The credibility of the study lay in the collection of data from a heterogeneous group of participants, meticulous transcription, and rigorous analysis of data by the research team. The transferability of the results was guaranteed through the description of the study setting, detailed descriptions of the experiences, and sampling strategy. Finally, dependability and confirmability were ensured through a detailed audit of the methodological and analytical decisions made by the research team throughout the study.

### Ethical considerations

This study followed the Helsinki Declaration [37] and the Oviedo Convention [38] for research with human beings. This study was approved by an Ethics Committee. All participants read the information on the study, clarified any doubts with the principal investigator, and signed informed consent form and consent for audio recording. This is a mandatory procedure for research involving human subjects [39]. All participants were informed that they could withdraw from the study at any time without resulting in any future harm to them and that their participation was completely voluntary. In addition, they were informed that all data resulting from the research would only be available only to the research team [26]. Even though all the participants had a diagnosed mental health disorder, none of them presented symptoms which could negatively impact their self-determination. Thus, there was no need to implement safeguards and/or ethical considerations to protect the participants.

## Findings

The sample comprised eight patients diagnosed with mental health disorder. All participants had previous experiences with nurses, 87.50% (n = 7) in inpatient and ambulatory contexts and only 1 (12.50%) in a home context. As for schooling, 12.50% completed the 11th of schooling (n = 1), and one participant completed the 8th year of schooling (12.50%). Table 1 details the characterization of the participants.

**Table 1:** Characterization of the participants

Variables		n	%
**Sex**	Male	4	50
	Female	4	50
**Academic Degree**	Bachelor’s Degree	2	25
	Secondary Education	4	50
	Other	2	25
**Age**	Maximum	55	
	Minimum	32	
	Average	42.50 (SD = 7.13)	
**Mental Health Disorder**	Schizophrenic Psychosis	6	75
	Schizoaffective Psychosis	2	25

Three themes emerged from the questions in the focus group (Table 2).

**Table 2:** Themes, categories, and codes obtained from content analysis

Themes	Definition	Categories	Codes
Theme 1	Behaviours in caregiving	Help Behaviour	Support
			To help
		Care Management/Organisation	Person surveillance
			Assessment of the person
			Provision of care depending on the patient’s dependence
		Patient’s Involvement in Care Decision-Making	Ask for an opinion before the intervention
			Explain the treatment and options
		Personalisation of Care	Ask about preferences
			Consider the patient and not the disorder
Theme 2	Attitudes in caregiving	Acceptance and Empathy	Understanding
		Kindness	Pleasant
			Sympathy
		Authenticity	Genuine
		Tranquillity	Relaxed
			Calm
		Respect	Address the patient by preferred name
		Availability	Dedicate time to the patient
		Setting Boundaries	Distancing in the relationship
Theme 3	Communication in caregiving	Verbal Communication	Tone of voice
			Speech speed
		Non-verbal Communication	Touch
			Presence

### Theme 1: behaviours in caregiving

In this domain of results, participants focused on a set of actions that they expect nurses to be able to perform through the therapeutic relationship, responding to the first questions of the questioning route used in the focus group meetings.

The participants’ expected the relationship they established was a facilitator of nurses’ support - *P1: “because we need to feel supported, the most important is that [the nurse] gives us their support*.” On the other hand, the participants also valued the ability of nurses to help the patient feel better and to recover - *P3: “they help us get better and recover*”, being this a collaborative process - *P5: “It’s very important to establish a mutual and reciprocal help relationship between patients and nurses*”. On the other hand, participants reported that the nurse should be able to assess and identify the patients’ problems/needs - *P4*: “*because it’s them who assess us since when we arrive to the inpatient unit the first persons we see are the nurses, who evaluate us depending on their training, they assess what we need”*; *P5*: “*the nurse is able to evaluate the patient and then discuss that with the physician and family, to administer medication or do other things. The nurse is the first to identify the patient’s problem because this is the main nurse’s work”*. After identifying needs, the participants considered that nurses should establish priorities. For example, care delivery should be prioritized according to the patient’s previous assessment - *P4: “nurses are concerned with everyone equally, but it depends on each case. Those who are bedridden need the most support, so they will focus on those patients first. I’m not saying that they stop worrying with the others, but they worry most about patients with more needs”*.

In addition, participants reported that nurses should acknowledge them when delivering care, for example, by asking their opinion about specific interventions - *P4: “we have to accept our disease and treatment. I like to give my opinion because this is my experience; I’m the one dealing with the disease”* or ask about their preferences - *P5: “since there is specific food for diabetic patients, the bathing time should be chosen by the patient”*.

Finally, participants highlighted that they should be addressed by their favourite names, so nurses should always ask this question. Also, participants considered that nurses should always introduce themselves. According to the findings, participants concluded that this assumption would be a facilitator of the relationship.

### Theme 2: attitudes in caregiving

A single category emerged from this theme, “attitudes in caregiving”.

These results emerged from the questions concerning the appropriate nurses’ attitudes as a facilitator of a good relationship with patients. Participants reported that they expected nurses to *P1:“be empathetic”, P8:“someone caring”, P4: “be calm”*, that shows respect for the patient - *P6: “it’s important that nurses treat us as patients, be respectful”, P5: “be respectful to the patient to be respected as well”*, be available for the patient - *P5: “The nurse is better if he/she dedicates more time to me”* and, finally, that the nurse is able to set boundaries in the relationship roles - *P8: “I think it’s important to keep a certain distance, be neutral so that each can do their work”*, *P4: “by doing so, nurses can still be professionals and - friends are friends, business aside -”.*

### Theme 3: communication in caregiving

The last theme focused on communication in caregiving, with participants referring to aspects related to verbal and non-verbal communication. They also emphasized the tone of voice as fundamental to establishing a good relationship with the nurse - *P7, P3: “nurses shouldn’t scream”, P1: “nurses should use a softer tone of voice”*. Moreover, the nurse’s speech speed was highlighted - *P6: “they should speak calmly and slowly”.* As for the non-verbal communication, participants highlighted touch as an important aspect of interaction - *P5: “it’s important that nurses use touching so that we feel safe”, P1: “it’s quite positive that nurses use touching”, P3: “touch is important because it reveals affection and attention”.* Additionally, listening to the patient was considered vital to a good relationship with the nurse - *P5: “it’s better for a patient to vent his/her feelings than taking pills”, P7: “when we’re sad, sometimes it’s best to express our emotions to someone willing to listen”, P5: “I feel quite well when speaking to the nurses, I feel relieved because someone is actually listening to me”.*

## Discussion

This study aimed to explore the perspectives of adult patients with mental health disorders on the relationship with nurses. The results focused on what is expected from the relationship with nurses, their expected skills, attitudes, and communications ways to facilitate a quality therapeutic relationship.

It is important to note that this study’s participants were patients with mental health disorders of the psychotic spectrum. The literature is consistent about the importance of involving patients with mental health disorder in the research, policy-making, and healthcare processes since they experience both the disease and the treatment [40]. Therefore, this study and its results meet the recommendations found in the literature about the importance of involving patients with mental health disorders in research processes. However, patients with psychotic spectrum disorders may present some additional vulnerability to therapeutic breakdowns or drawbacks since it is common for this group of patients to have a delusional understanding of the context or lack of insight regarding their clinical situations [41].

This study highlights the importance that patients with mental health disorders attribute to their relationship with nurses, hoping that they will highly contribute to the patient’s recovery process. This is in line with the literature since, through this relationship, a helping behaviour is expected to promote a change in the patient’s behaviour, aiming at a better improvement and recovery [42]. However, the literature supports this premise centered on the nurse’s action and it is unknown whether this is also the same expectation of patients with mental health disorders. Thus, the results of this study enhance the perception of the recipients of nursing care being aligned with what is expected from the nurse’s action.

On the other hand, the results showing the identification of the individual needs of each patient and the response to those needs are also corroborated by the literature. Thus, in a study conducted by Girmay [43] with 281 patients (without mental health disorder), these aspects were identified by participants as expected from the nurse’s relationship and care [43]. In addition, the patient’s involvement in decision-making regarding the provision of care was an identified result. Importantly, the relationship between the patient and the nurse is expected to be non-hierarchical, with all the elements involved working equally toward a common purpose [44, 45]. According to the results, decision-making in care should be shared on the one hand because it promotes a patient-centred approach and recognizes the patient’s right to autonomy and self-determination [44]. However, on the other hand, patients who seek information and want to make their own decisions are often seen as “bad patients” [46]. Thus, studies involving participants with mental health disorder are important to deconstruct this idea. The results of this study should encourage the involvement of patients with mental health disorders in the design of their treatment plan, countering the existing traditional imbalance, which focuses on health professionals in decision-making [44]. The findings of this work are also useful in refuting the paradigm of the limitations of patients with mental health disorders in decision-making about their care, as described in the study by Haugom et al. [47].

This study stressed the concern with the personalisation of the relationship and care. According to the literature, care personalisation is a way to improve the care provided [48]. Thus, these study results are in line with the existing literature. For example, concerning patients with depressive and/or anxiety disorders, when care is personalised, they are involved in discussions about their problems and in setting their priorities and an action plan that promotes their well-being [49]. Notably, this is not a new concept since, in 1963, a study with 48 patients with psychosis receiving individual and personalised nursing care showed a significant improvement in their health status [50].

As for the nurses’ skills/attitudes identified in this study, acceptance, empathy, availability, authenticity, and respect are highlighted. Overall, these findings are in line with the literature. Thus, for example, in a study conducted with 12 participants with borderline personality disorder, nurses’ attitudes were pointed out as essential for the relationship and trust, such as being non-judgemental, availability, and humanity [51]. Also, about nurses’ attitudes, one of the participants in this study referred to genuineness. Again, the literature shows that being genuine promotes solid relationships [52]. Moreover, Carl Rogers argues that genuineness and honesty are crucial for consistent relationships, as respect for the ill patient [53]. Concerning empathy, the literature shows that nurses have the highest levels of empathy and the best attitudes towards patients with mental health disorder [54], signalling empathy as most expected from nurses [55]. In a study conducted with 285 inpatients, empathy was also highlighted as a positive aspect expected from nurses to promote recovery [56].

Participants also mentioned verbal and non-verbal communication as crucial to their relationship with nurses. These results are in line with the literature that highlights communication as something central to the interaction between nurses and patients being crucial for achieving positive health outcomes [57–59]. In a qualitative study conducted with seven inpatients aiming at understanding the barriers to therapeutic communication between patients and nurses, the participants referred to the language and vocabulary used and the nurses’ limited availability to listen corroborating these study results [60].

Importantly, these study results highlight the importance of the relationship between the nurse and the patient with mental health disorder from the patient’s perspective. These findings also show that there is still much to do so that patients with mental health disorder can play a more active role and freely exercise their self-determination through this relationship and making this an excellent starting point for nurses to reflect on their skills, attitudes, and perceptions towards the relationship with patients with mental health disorder.

This is an innovative study because it was developed with patients with mental health disorder who were actively engaged in the research. The purpose is to provide direct recipients of nursing care with the opportunity to be included and actively involved in the therapeutic relationship.

The results of this study may be relevant for the humanization of care since knowing the perspectives of patients with mental health disorders helps create more welcoming environments sensitive to individual needs and facilitators in improving the quality and safety of care. On the other hand, adherence to the therapeutic regimen is also enhanced, which is crucial for maintaining the well-being of patients with mental health disorders and good management of health resources and services. Understanding the needs and expectations of patients with mental health disorders allows managers to adjust resources and policies to better meet the demands of the population, optimizing the allocation of resources. Finally, this work helped to raise awareness about the need to involve patients with mental health disorders in their recovery process, co-production in care, and political inclusion [61].

This study had one important limitation related to the fact that all the participants had mental health disorders only of the psychotic spectrum. Notwithstanding this limitation, it is essential to understand which factors represent a good therapeutic relationship with the nurse from the perspective of patients with mental health disorder within the psychotic spectrum so that it can be improved and targeted [62, 63]. However, future studies should consider patients with mental health disorders of a different spectrum.

### Relevance for clinical practice

Notably, these study findings are relevant for nursing practice, such as mental health nursing, since they highlight the importance of the relationship with nurses from the patient’s perspective and to what extent this is relevant to their recovery process. Especially for mental health nurses, the exercise of self-reflection and self-knowledge is essential to the quality of their clinical practice. Therefore, these study results are beneficial to this reflective process since, through this process, nursing professionals may improve their relational and communicational skills. On the other hand, the results of this study may be the starting point for future research, for example, the development of instruments to assess the relationship between the patient and the nurse from the patient’s perspective.

Finally, this study will elevate nursing education since the participants’ direct participation throughout the study will promote students’ awareness of the relevance of the therapeutic relationship.

## Conclusion

In this study, participants considered the relationship with nurses relevant to their recovery process, expecting them to provide help, support, and assistance by showing empathy, acceptance, understanding, and respect for the other. Communication was also highlighted as pivotal for establishing a good relationship between both parties.

Nursing care is expected to be patient-centred, involving the patient in decision-making and understanding his/her expectations, values, and preferences. This will add true meaning to the relationship between patient and nurse, enhancing quality and promoting the patient’s recovery.

## Data Availability

The datasets used and/or analysed during the current study are available from the corresponding author on reasonable request.

## Abbreviations

COREQ: Consolidated Criteria for Reporting Qualitative Research
